# Evaluating pegenzileukin (SAR444245/THOR-707) in combination with pembrolizumab or cetuximab in advanced metastatic gastrointestinal cancer: the PEGATHOR phase 2 study

**DOI:** 10.1007/s10637-026-01622-2

**Published:** 2026-06-22

**Authors:** William P. Harris, Pilar García-Alfonso, Jean Phillipe Metges, Antonio Cubillo-Gracian, Aziz Zaanan, Lopez Lopez Carlos, Monica Ronzoni, Mariano Ponz-Sarvise, Adyb Baakili, Asma Kefsi, Laurent Andrieu, Maria Agrawal, Wenting Wang, Tobias Paehler, Giovanni Abbadessa, Eric Van Cutsem

**Affiliations:** 1 University of Washington Medical Center, Fred Hutchinson Cancer Center, Seattle, WA USA; 2 Medical Oncology Department, Hospital General Universitario Gregorio Marañón, Instituto de Investigación Sanitaria Gregorio Marañón (IiSGM), Universidad Complutense, Madrid, Spain; 3 Institut de Cancérologie et d’Imagerie CHU Cavale Blanche ARPEGO NETWORK, Brest, France; 4 HM CIOCC MADRID (Centro Integral Oncológico Clara Campal), Hospital Universitario HM Sanchinarro, HM Hospitales, Madrid, Spain; 5 Facultad HM de Ciencias de La Salud de La Universidad Camilo José Cela, Madrid, Spain; 6 Instituto de Investigación Sanitaria HM Hospitales, Madrid, Spain; 7 Department of Digestive Oncology, Georges Pompidou European Hospital, Assistance Publique - Hôpitaux de Paris, University of Paris Cité, Paris, France; 8 Hospital Universitario Marques de Valdecilla, IDIVAL, UNICAN, Santander, Spain; 9 Vita-Salute San Raffaele University, Milan, Italy; 10 Medical Oncology Department, IRCCS San Raffaele Scientific Institute, Milan, Italy; 11 Department of Medical Oncology and Program in Solid Tumors, Cima Universidad de Navarra, Cancer Center Clínica Universidad de Navarra (CCUN), Pamplona, Spain; 12 Sanofi-Aventis Recherche & Développement, Paris, France; 13 Sanofi, Cambridge, MA USA; 14 Sanofi-Aventis Deutschland, Frankfurt, Germany; 15 University Hospitals Gasthuisberg, University of Leuven, Leuven, Belgium

**Keywords:** Pegenzileukin, SAR444245, PEGATHOR, Gastrointestinal cancer, Pembrolizumab, Cetuximab, Interleukin-2

## Abstract

Pegenzileukin (SAR444245) is a pegylated recombinant human IL-2 variant designed to expand effector T cells and NK cells without stimulating regulatory T cells. This study evaluated pegenzileukin with pembrolizumab or cetuximab for treating advanced gastrointestinal cancers. A total of 138 patients with advanced gastric cancer (GC), colorectal carcinoma, esophageal squamous cell carcinoma (ESCC), and hepatocellular carcinoma (HCC) were enrolled across seven cohorts in this phase 2 study. Patients received pegenzileukin (24 µg/kg every 3 weeks [Q3W]) with pembrolizumab (200 mg Q3W) or cetuximab (initially 400 mg/m^2^ and then 250 mg/m^2^ weekly). The primary endpoint was the objective response rate (ORR). The secondary endpoints were time to response, response duration, clinical benefit rate, progression-free survival (PFS), and safety. Exploratory biomarker analyses assessed immune cell expansion and cytokine levels. The ORR varied across the cohorts, with the highest in ESCC cohort (1/5; 20.0%) and the lowest in GC Cohort 2 and the HCC cohort (1/19; 5.3% and 1/20; 5.0%, respectively). The median PFS was consistent across the cohorts (1.9–2.1 months). The most common treatment-emergent adverse events were asthenia (Pegenzileukin + pembrolizumab) and infusion-related reactions (Pegenzileukin + cetuximab). The biomarker analyses demonstrated robust expansion of CD8^+^ T cells, CD8^+^ Ki67^+^ T cells, NK cells, and NK Ki67^+^ cells without significant modulation of regulatory T cells. Pegenzileukin with pembrolizumab or cetuximab demonstrated limited efficacy, with a manageable safety profile in advanced GI cancers. The biomarker data supported the mechanism of action involving the upregulation of CD8^+^ T cells and NK cells. ClinicalTrials.gov Identifier: NCT05104567.

**Supplementary Information:**

The online version contains supplementary material available at https://doi.org/10.1007/s10637-026-01622-2.

## Introduction

Gastrointestinal (GI) cancers, such as colorectal carcinoma (CRC), esophageal squamous cell carcinoma (ESCC), gastric cancer (GC), hepatocellular carcinoma (HCC), and gastro-esophageal junction (GEJ) adenocarcinoma, are among the most prevalent cancers worldwide [30]. Advanced stages of these cancers often involve metastasis, resulting in a very poor prognosis, especially after progression despite initial standard therapies. The 5-year relative survival rates for metastatic stages are approximately 16.3% for CRC [1], 5% for ESCC [12], 7% for GC [2], 2% for HCC [10], and < 5% for GEJ adenocarcinoma [6], emphasizing the urgent need for improved treatment strategies.

The standard of care for GI cancers encompasses multimodal approaches tailored to the specific type and stage of cancer. Approved therapies include chemotherapy regimens (e.g., FOLFOX and FOLFIRI) and monoclonal antibodies, such as antiangiogenics (bevacizumab) or anti-EGFR agents (cetuximab or panitumumab), for colorectal cancer [28], chemotherapy combined with targeted therapies, such as trastuzumab, for human epidermal growth factor receptor (HER) 2-positive GCs [5], and immune checkpoint inhibitor-based therapies or multi-kinase inhibitors (e.g., nivolumab/ipilimumab, durvalumab/tremelimumab, sorafenib, and lenvatinib) or combinations of antiangiogenic monoclonal antibodies (atezolizumab plus bevacizumab) for advanced HCC [20, 31]. For ESCC, immunotherapy combinations with platinum-based chemotherapy have emerged as the standard first-line treatment, with pembrolizumab plus chemotherapy demonstrating improved survival outcomes in the KEYNOTE-590 trial [26]. However, treatment strategies are increasingly personalized based on cancer subtype, molecular profile, and disease stage.

Immunotherapy has significantly advanced the treatment of GI cancers. Immune checkpoint inhibitors targeting cytotoxic T-lymphocyte antigen-4 and programmed cell death-1 (PD1) have significantly advanced the treatment of GI cancers. These inhibitors function by blocking T-cell inactivation and enhancing the antitumor immune response [17]. PD-1 inhibitors have demonstrated efficacy in various GI cancers. Pembrolizumab is approved for microsatellite instability-high/mismatch repair deficient (MSI-H/dMMR) metastatic colorectal cancer and in combination with trastuzumab, fluoropyrimidine- and platinum-containing chemotherapy as a first-line treatment for PD-L1-positive gastric or gastro-esophageal junction (GEJ) [22] and ESCC [26]. Nivolumab in combination with ipilimumab has recently been approved as a first-line treatment for patients with MSI-H/dMMR metastatic colorectal cancer [3].

Nivolumab in combination with fluoropyrimidine- and platinum-containing chemotherapy is approved for treating advanced or metastatic GC, GEJ cancer, esophageal adenocarcinoma, and ESCC a whose tumors express PD-L1 (≥ 1) [7, 13]. However, its response rates vary, ranging from 30 to 60% in MSI-H/dMMR colorectal cancer and from 10 to 15% in microsatellite stable tumors [18, 23]. Furthermore, the combination of tremelimumab and durvalumab is approved as the first-line treatment for adult patients with unresectable or metastatic HCC [4].

Recombinant human interleukin (IL)−2 (aldesleukin) received approval in 1992 in the United States for treating renal cell carcinoma and later in 1998 for treating metastatic melanoma [25]. High-dose IL-2, the first approved cancer immunotherapy, has limited efficacy because it stimulates regulatory T cells (Treg), thereby reducing immune responses, and causes significant side effects, such as vascular leak syndrome and cytokine release syndrome [25]. Thus, a pressing need exists for novel immunotherapies to overcome resistance mechanisms and improve patient outcomes while maintaining tolerable safety profiles.

Pegenzileukin (SAR444245/THOR-707) is a pegylated recombinant human IL-2 variant designed to prevent IL-2Rα engagement while retaining IL-2Rβγ interaction, hence promoting effector T-cell and NK-cell expansion without stimulating regulatory T cells. Pegenzileukin enhanced the antitumor effects of aldesleukin in animal models, both as a single agent and in combination with anti-PD1 therapy, without severe side effects [15]. The interim analysis of the HAMMER trial demonstrated initial efficacy and a tolerable safety profile for pegenzileukin, both as a monotherapy and in combination with pembrolizumab, in advanced and metastatic solid tumors [15]. PEGATHOR, a phase 2, non-randomized, open-label, multicohort, multicenter study, assessed the potential clinical benefit of pegenzileukin in combination with other anticancer therapies for the treatment of patients with advanced unresectable or metastatic skin cancer (NCT04913220), lung cancer or pleural mesothelioma (NCT04914897), advanced and metastatic GI cancer (NCT05104567), head and neck squamous cell carcinoma (NCT05061420), and relapsed or refractory B-cell lymphoma (NCT05179603).

The present study reports the results from a subgroup of patients with advanced GI cancer, wherein the efficacy, safety, and biomarker data of pegenzileukin in combination with other anticancer therapies (pembrolizumab or cetuximab) for the treatment of advanced and metastatic GI cancer were assessed.

## Methods

### Study oversight

This study was conducted in accordance with the Good Clinical Practice guidelines established by the International Council for Harmonisation of Technical Requirements for Pharmaceuticals for Human Use and the ethical principles outlined in the Declaration of Helsinki. Written informed consents were obtained from all patients prior to the study procedure. The protocols, amendments, and informed consents received appropriate approval from the Institutional Review Board prior to the initiation of the study. The study was registered on ClinicalTrials.gov (NCT05104567). Clinical trial data can be accessed from ClinicalTrials.gov with study id number ACT16902. The study was proceeded according to the protocol guidelines.

### Study design and patients

This was a phase 2, multicohort, uncontrolled, non-randomized, open-label, multicenter study conducted at 29 active centers across 11 countries (the USA, Spain, Italy, South Korea, Belgium, France, Poland, the Netherlands, China, Germany, and Chile). The study included seven cohorts, initially planned to assess pegenzileukin in combination with pembrolizumab or cetuximab in patients with advanced or metastatic GI cancer. An overview of the study interventions and disease indications for each cohort is provided in Table S1. The cohorts were classified based on cancer type (GC, CRC, ESCC, and HCC), the line of therapy, and patients’ PD-1 status.

Patients were 18 years of age with histologically or cytologically confirmed diagnoses of GC or Siewert Type 2 and 3 GEJ adenocarcinoma (cohorts 1–3), CRC (cohorts 4 and 5), ESCC (cohort 6), and HCC (cohort 7). Patients had at least one measurable lesion as per RECIST 1.1 criteria [8]. Female patients must not be pregnant or breastfeeding and, if of childbearing potential, must use contraception. Male patients had to refrain from sperm donation and utilize contraception throughout the study treatment. Patients who had an Eastern Cooperative Oncology Group (ECOG) performance status (PS) score of ≥ 2, a predicted life expectancy of ≤ 3 months, a history of allogenic tissue or solid organ transplant, active brain metastases or leptomeningeal disease, recent antitumor therapy (within 28 days or less than five times the half-life, whichever was shorter), a major surgery or local intervention within 28 days, a comorbidity requiring corticosteroid therapy (> 10 mg prednisone/day), recent antibiotic use (≤ 14 days before the start of the therapy), a severe cardiac condition within the last 6 months, an active autoimmune disease, or a second malignancy were excluded.

A graphical presentation of the study schema is depicted in Supplementary Fig. S1. Pegenzileukin was administered at a dose of 24 µg/kg via a 30-min intravenous (IV) infusion every 3 weeks (Q3W) on day 1 of each 21-day cycle, for up to 35 cycles or until the occurrence of progressive disease (PD), unacceptable adverse events (AEs), or other criteria requiring permanent discontinuation. Pembrolizumab was administered at a dose of 200 mg via a 30-min IV infusion on day 1 of each 21-day cycle, for up to 35 cycles. Cetuximab was administered with an initial loading dose of 400 mg/m^2^ over 120 min on cycle 1 day 1, followed by 250 mg/m^2^ over 60 min for all subsequent doses starting on cycle 1 day 8, until PD. This study was terminated on October 21, 2022, for non-safety reasons. By this time, all cohorts had been initiated.

### Study assessments

The primary efficacy endpoint included the objective response rate (ORR), which encompassed a confirmed complete response and a partial response (PR). The secondary efficacy endpoints included the time to response (TTR), the duration of response (DOR), the clinical benefit rate (CBR), and progression-free survival (PFS). The PFS was defined as the time from the first drug administration to the date of the first documented disease progression determined by the investigator as per RECIST 1.1 or death from any cause, whichever occurred first.

The incidences of treatment-emergent adverse events (TEAEs), laboratory abnormalities, and serious adverse events (SAEs) were assessed as per the National Cancer Institute Common Terminology Criteria for Adverse Events (NCI CTCAE) V5.0 and the American Society for Transplantation and Cellular Therapy (ASTCT) guidance.

### Biomarker analysis

Tumor biopsy and peripheral blood samples were collected during the study for exploratory biomarker analyses to assess target engagement and the immune modulatory effects of pegenzileukin in combination with pembrolizumab/cetuximab.

Blood samples for immune cell phenotyping were collected at specified time points throughout the study. Flow cytometry was used to evaluate changes in immune cell subsets, including CD8^+^ T cells, Treg cells, and NK cells. The blood levels of pro-inflammatory cytokines and soluble factors, such as tumor necrosis factor (TNF)-α, IL-6, IL-5, IL-8, and interferon γ, were assessed at specified time points. Tumor tissue was analyzed for changes in infiltrating immune cells (CD8^+^ T cells and Treg cells) and immune markers (PD1) using multiplex immunofluorescence imaging-based methods.

### Statistical analysis

A target sample size of approximately 40 patients from each cohort were used for study planning purposes. The efficacy population included all patients who had received at least one dose of pegenzileukin, pembrolizumab, or cetuximab, had at least one evaluable tumor assessment post baseline, or had permanently discontinued the study treatment.

The ORR and the best overall response were summarized for the efficacy population using descriptive statistics. A two-sided 90% confidence interval was computed using the Clopper–Pearson method. The TTR, DOR, and CBR were assessed in patients who achieved a confirmed objective response in the efficacy population. The PFS was summarized using the Kaplan–Meier method. The safety and biomarker results were summarized for all patients using descriptive statistics.

Boxplots with data points and lines were utilized to visualize the distribution of immune cells at baseline and peak post-treatment, highlighting the change in trend. The center of the box represents the median, whereas the upper and lower lines indicate the upper and lower quartiles, respectively. The vertical lines extending from the box denote 1.5 times the interquartile range. A one-sided paired *t*-test was conducted to assess the significance of immune cell expansion at peak post-treatment.

### Data availability

Qualified researchers may request access to patient-level data and related documents [including, e.g., the clinical study report, study protocol with any amendments, blank case report form, statistical analysis plan, and dataset specifications]. Patient-level data will be anonymized, and study documents will be redacted to protect the privacy of trial participants. Further details on Sanofi's data sharing criteria, eligible studies, and process for requesting access can be found at https://www.vivli.org. 

## Results

### Study population

A total of 138 patients were enrolled across all cohorts: 22 in cohort 1, 19 in cohort 2, 18 in cohort 3, 30 in cohort 4, 24 in cohort 5, 5 in cohort 6, and 20 in cohort 7. All enrolled patients received treatment as predefined in the protocol. The main reason for discontinuation was PD, which was observed in 90 (76.3%) patients. As of the database lock on October 6, 2023, three (2.5%) patients were still receiving the study intervention. Patients’ disposition for each cohort is provided in Supplementary Table S2.

The mean age was comparable across all cohorts, ranging from 58 to 66 years, with 50–73% of patients aged under 65 years. The cohorts were predominantly comprised of male and white patients, with the majority having an ECOG PS score of 0 and 1. The majority of patients had stage 4 disease and had received two or more prior therapies. Detailed demographic and baseline characteristics are presented in Table 1.

**Table 1:** Demographic and baseline characteristics

	Cohort 1 Pegenzileukin 24 µg/kg + pembrolizumab (***N*** = 22)	Cohort 2 Pegenzileukin 24 µg/kg + pembrolizumab (***N*** = 19)	Cohort 3 Pegenzileukin 24 µg/kg + pembrolizumab (***N*** = 18)	Cohort 4 Pegenzileukin 24 µg/kg + pembrolizumab (***N*** = 30)	Cohort 5 Pegenzileukin 24 µg/kg + cetuximab (***N*** = 24)	Cohort 6 Pegenzileukin 24 µg/kg + pembrolizumab (***N*** = 5)	Cohort 7 Pegenzileukin 24 µg/kg + pembrolizumab (***N*** = 20)
Age (years), mean (SD)	58.1 (10.3)	56.5 (13.1)	58.3 (10.9)	62.7 (12.1)	61.7 (10.0)	63.2 (4.4)	65.7 (8.8)
Male, ***n*** (%)	17 (77.3)	12 (63.2)	14 (77.8)	19 (63.3)	17 (70.8)	3 (60.0)	14 (70.0)
Race, ***n*** (%)							
White	13 (59.1)	15 (78.9)	10 (55.6)	30 (100)	18 (75.0)	3 (60.0)	14 (70.0)
Black or African American	0	0	0	0	1 (4.2)	0	0
Asian	1 (4.5)	2 (10.5)	6 (33.3)	0	4 (16.7)	0	3 (15.0)
Not reported	6 (27.3)	1 (5.3)	1 (5.6)	0	1 (4.2)	2 (40.0)	3 (15.0)
Unknown	2 (9.1)	1 (5.3)	0	0	0	0	0
BMI (kg/m^2^), mean (SD)	22.4 (3.8)	23.1 (3.6)	22.3 (3.9)	24.9 (4.4)	24.6 (5.3)	26.8 (7.2)	24.8 (4.4)
ECOG PS, ***n*** (%)							
0	10 (45.5)	8 (42.1)	3 (16.7)	10 (33.3)	8 (33.3)	3 (60.0)	13 (65.0)
1	12 (54.5)	11 (57.9)	15 (83.3)	20 (66.7)	15 (62.5)	2 (40.0)	6 (30.0)
2	0	0	0	0	1 (4.2)	0	1 (5.0)
Time from the initial diagnosis to the first pegenzileukin administration							
Median years (Min–Max)	1.2 (0–5) (*n* = 22)	1.3 (0–8) (*n* = 19)	1.5 (0–5) (*n* = 18)	3.1 (1–9) (*n* = 29)	3.2 (1–10) (*n* = 22)	3.2 (1–5) (*n* = 5)	2.7 (1–10) (*n* = 20)
Extent at the study entry, ***n*** (%)							
Metastatic	19 (86.4)	19 (100)	16 (88.9)	30 (100)	24 (100)	3 (60.0)	12 (60.0)
Locally advanced	3 (13.6)	0	2 (11.1)	0	0	2 (40.0)	8 (40.0)
Number of organs involved, ***n*** (%)							
1	4 (18.2)	2 (10.5)	1 (5.6)	2 (6.7)	4 (16.7)	2 (40.0)	7 (35.0)
2	8 (36.4)	8 (42.1)	6 (33.3)	11 (36.7)	7 (29.2)	1 (20.0)	7 (35.0)
3	4 (18.2)	4 (21.1)	4 (22.2)	11 (36.7)	6 (25.0)	1 (20.0)	5 (25.0)
> 3	6 (27.3)	5 (26.3)	7 (38.9)	6 (20.0)	7 (29.2)	1 (20.0)	1 (5.0)
Number of prior lines in advanced settings, ***n*** (%)							
0	5 (22.7)	2 (10.5)	0	0	0	0	2 (10.0)
1	5 (22.7)	10 (52.6)	6 (33.3)	2 (6.7)	1 (4.2)	3 (60.0)	7 (35.0)
2	11 (50.0)	6 (31.6)	3 (16.7)	10 (33.3)	8 (33.3)	2 (40.0)	11 (55.0)
3	1 (4.5)	1 (5.3)	9 (50.0)	8 (26.7)	6 (25.0)	0	0
> 3	0	0	0	10 (33.3)	9 (37.5)	0	0
Type of prior anticancer^a^ therapy, ***n*** (%)							
Number of prior anticancer therapy	1 (4.5)	0	0	0	0	0	0
Chemotherapy	21 (95.5)	18 (94.7)	17 (94.4)	30 (100)	24 (100)	5 (100)	3 (15.0)
Hormonotherapy	0	0	0	0	0	0	0
Immunotherapy	1 (4.5)	0	17 (94.4)	0	0	5 (100)	19 (95.0)
Targeted therapy	5 (22.7)	8 (42.1)	11 (61.1)	29 (96.7)	24 (100)	0	19 (95.0)
Other	1 (4.5)	0	0	8 (26.7)	5 (20.8)	0	1 (5.0)

*BMI* body mass index, *SD* standard deviation

^a^A patient could be counted in several categories

In GC cohorts 1, 2, and 3 (*N* = 59), the median duration of exposure to pegenzileukin and pembrolizumab was 2.1 months (range 1–15 months), with 62.7% treated for at least three cycles. In CRC cohort 4 (*N* = 30), the median duration was 2.1 months (range: 1–8 months), with 40.0% treated for at least four cycles. In CRC cohort 5 (*N* = 24), the median duration was 3.8 months (range 1–8 months), with 50.0% treated for at least five cycles. In ESCC Cohort 6 (*N* = 5), the median duration was 3.4 months (range 2–8 months), with all patients treated for at least three cycles. In HCC cohort 7 (*N* = 20), the median duration was 2.1 months (range 1–15 months), with 75.0% treated for at least three cycles.

### Efficacy

#### Primary efficacy endpoint

Across all cohorts, PRs were observed in 10/138 patients, with the notable response rate in ESCC cohort 6 (1/5; 20.0%) and the lower in GC cohort 2 and HCC cohort 7 (1/19; 5.3% and 1/20; 5.0%, respectively). Stable disease was achieved in 34/138 patients, with the highest occurrence in CRC Cohort 5 (8/24; 33.3%) (Table 2).

**Table 2:** Efficacy of pegenzileukin in combination with pembrolizumab or cetuximab in patients with metastatic GI cancer

	Cohort 1 Pegenzileukin 24 µg/kg + pembrolizumab (***N*** = 22)	Cohort 2 Pegenzileukin 24 µg/kg + pembrolizumab (***N*** = 19)	Cohort 3 Pegenzileukin 24 µg/kg + pembrolizumab (***N*** = 18)	Cohort 4 Pegenzileukin 24 µg/kg + pembrolizumab (***N*** = 30)	Cohort 5 Pegenzileukin 24 µg/kg + cetuximab (***N*** = 24)	Cohort 6 Pegenzileukin 24 µg/kg + pembrolizumab (***N*** = 5)	Cohort 7 Pegenzileukin 24 µg/kg + pembrolizumab (***N*** = 20)
Best overall response, ***n*** (%)							
Complete response^a^	0	0	0	0	0	0	0
Partial response^a^	3 (13.6)	1 (5.3)	2 (11.1)	0	2 (8.3)	1 (20.0)	1 (5.0)
Stable disease	5 (22.7)	4 (21.1)	3 (16.7)	7 (23.3)	8 (33.3)	3 (60.0)	4 (20.0)
Progressive disease	12 (54.5)	10 (52.6)	12 (66.7)	23 (76.7)	14 (58.3)	1 (20.0)	14 (70.0)
Non-evaluable	2 (9.1)	4 (21.1)	1 (5.6)	0	0	0	1 (5.0)
Objective response rate (confirmed CR and PR), ***n*** (%)	3 (13.6)	1 (5.3)	2 (11.1)	0	2 (8.3)	1 (20.0)	1 (5.0)
90% CI^b^	(3.8–31.6)	(0.3–22.6)	(2.0–31.0)	(0.0–9.5)	(1.5–24.0)	(1.0–65.7)	(0.3–21.6)
Clinical benefit rate (confirmed CR, confirmed PR, and SD of at least 6 months), ***n*** (%)	5 (22.7)	2 (10.5)	2 (11.1)	2 (6.7)	3 (12.5)	2 (40.0)	2 (10.0)
90% CI^b^	(9.4–42.0)	(1.9–29.6)	(2.0–31.0)	(1.2–19.5)	(3.5–29.2)	(7.6–81.1)	(1.8–28.3)
	***N* = 3**	***N* = 1**	***N* = 2**	***N* = 0**	***N* = 2**	***N* = 1**	***N* = 1**
Time to response (months), mean	2.0	4.2	3.0	-	2.0	1.9	6.0

*CI* confidence interval, *CR* complete response, *SD* stable disease

^a^Confirmation of response was required (i.e., a second examination done at least 4 weeks apart); ^b^Estimated by the Clopper–Pearson interval

#### Secondary efficacy endpoints

The mean TTR varied across the cohorts. CRC cohort 5 showed the shortest mean TTR at 2.0 months (*n* = 2), whereas GC cohort 2 had the longest at 4.2 months (*n* = 1). The median DOR was the highest in GC cohort 1 at 13.1 months (*n* = 3), but it could not be calculated for cohorts 2, 6, and 7 due to a lack of events. The CBR also varied, with ESCC Cohort 6 achieving the highest CBR at 40.0% (2/5), whereas CRC cohort 4 had the lowest at 6.7% (2/30). These findings highlight the slight variability in response times and clinical benefits across different cohorts, as indicated in Table 2.

**Fig. 1:**
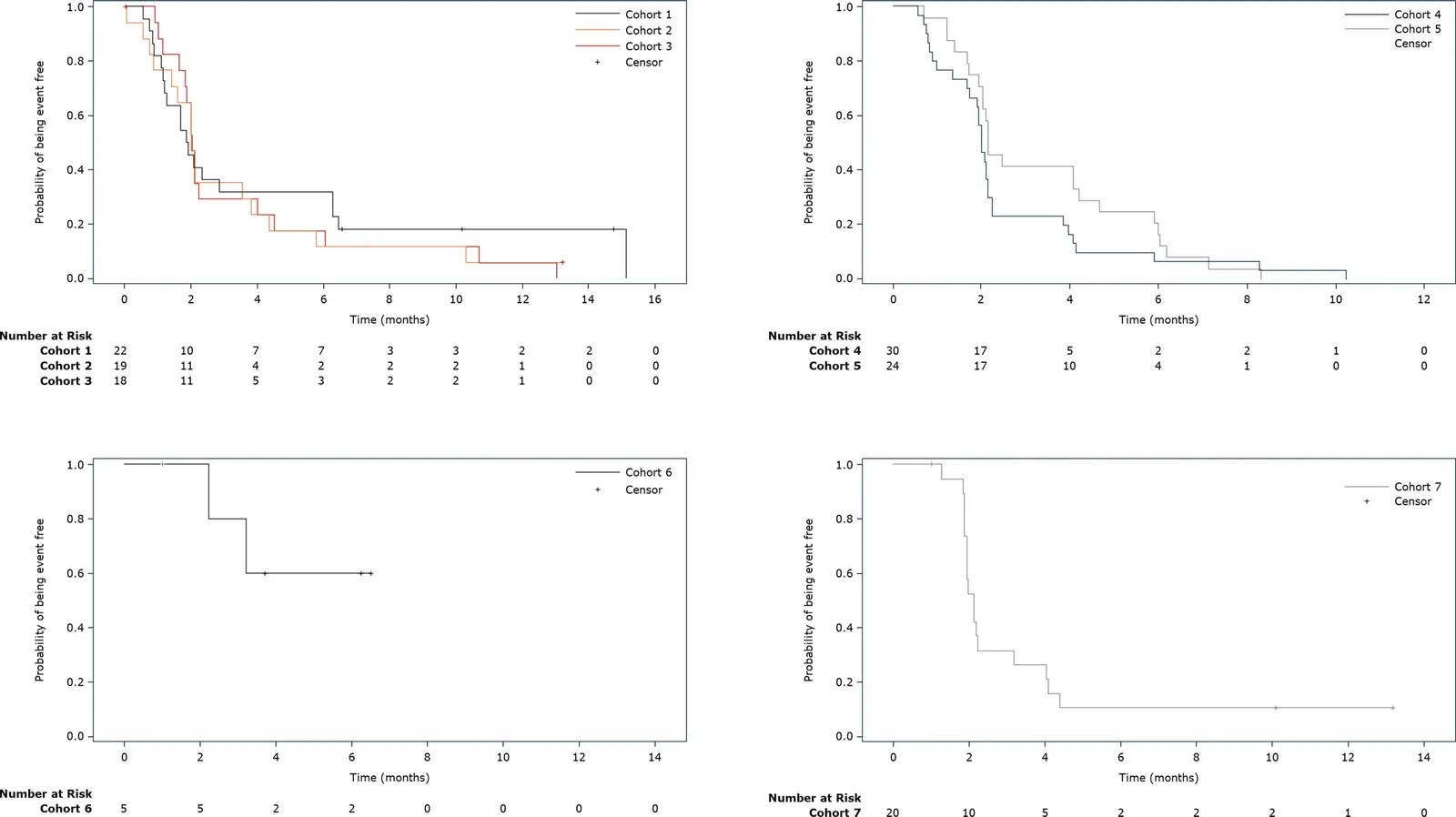
The Kaplan–Meier curve showing the progression-free survival of the GC (1–3), CRC (4 and 5), ESCC (6), and HCC (7) cohorts

The median PFS was consistent across the cohorts. In the GC cohorts (1–3), PFS ranged from 1.9 to 2.1 months. The CRC cohorts (4 and 5) showed similar results, with 2.0–2.1 months. The HCC cohort (7) had a median PFS of 2.1 months. For the ESCC cohort (6), the median PFS was incalculable due to insufficient events. Figure 1 illustrates the Kaplan–Meier curves for PFS.

### Safety

An overview of AEs is provided in Table 3 and Supplementary Table S3. The most frequently reported TEAE associated with pegenzileukin in combination with pembrolizumab was asthenia, occurring in 37.3% of patients in GC cohorts 1, 2, and 3; 53.3% in cohort 4 (CRC); 60.0% in ESCC cohort 6, and 45.0% in HCC cohort 7. In CRC cohort 5 (pegenzileukin + cetuximab), the most common TEAE was infusion-related reactions (45.8%). Common TEAEs related to pegenzileukin in combination with pembrolizumab (> 10%) included infusion-related reactions, asthenia, cytokine release syndrome, and diarrhea. In cohort 5 (pegenzileukin + cetuximab), common TEAEs (> 10%) included folliculitis, cytokine release syndrome, decreased appetite, nausea, vomiting, diarrhea, rash, pruritus, dry skin, dermatitis acneiform, asthenia, influenza-like illness, fatigue, and infusion-related reactions. In the pegenzileukin + pembrolizumab cohorts, eight patients experienced fatal AEs, including COVID-19, pneumonia, respiratory tract infection, hypovolaemic shock, small intestinal perforation, and cholestatic jaundice; of these eight patients, two exhibited adverse events of disease progression and six suffered events unrelated to disease progression and to study treatments. Seven (six in all GC cohorts 1–3 and one in CRC cohort 4) deaths were due to PD during the treatment-emergent period. In cohort 5 (pegenzileukin + cetuximab), five deaths were due to PD during the treatment-emergent period (Supplementary Table S4).

**Table 3:** Safety profile of pegenzileukin in combination with pembrolizumab or cetuximab in patients with metastatic GI cancer

*n* (%)	Cohort 1Pegenzileukin 24 µg/kg + pembrolizumab (*N* = 22)	Cohort 2 Pegenzileukin 24 µg/kg + pembrolizumab (*N* = 19)	Cohort 3 Pegenzileukin 24 µg/kg + pembrolizumab (*N* = 18)	Cohort 4 Pegenzileukin 24 µg/kg + pembrolizumab (*N* = 30)	Cohort 5 Pegenzileukin 24 µg/kg + cetuximab (*N* = 24)	Cohort 6 Pegenzileukin 24 µg/kg + pembrolizumab (*N* = 5)	Cohort 7 Pegenzileukin 24 µg/kg + pembrolizumab (*N* = 20)
Any TEAE	22 (100)	19 (100)	18 (100)	30 (100)	24 (100)	5 (100)	19 (95.0)
Any grade ≥ 3 TEAE	16 (72.7)	14 (73.7)	10 (55.6)	14 (46.7)	9 (37.5)	2 (40.0)	6 (30.0)
Any grade 5 TEAE^a^	8 (36.4)	5 (26.3)	1 (5.6)	1 (3.3)	5 (20.8)	0	0
Any treatment-related TEAE	17 (77.3)	16 (84.2)	16 (88.9)	29 (96.7)	24 (100)	5 (100)	19 (95.0)
Any grade ≥ 3 treatment-related TEAE	3 (13.6)	5 (26.3)	5 (27.8)	7 (23.3)	1 (4.2)	0	4 (20.0)
Any TEAE leading to permanent full discontinuation of intervention	5 (22.7)	3 (15.8)	0	2 (6.7)	0	0	3 (15.0)
Any TEAE leading to permanent partial discontinuation of intervention							
Pegenzileukin	0	0	2 (11.1)	0	1 (4.2)	0	0
Pembrolizumab	0	0	0	0	0	0	0
Cetuximab	0	0	0	0	0	0	0
Any treatment-emergent SAE	17 (77.3)	13 (68.4)	9 (50.0)	19 (63.3)	11 (45.8)	2 (40.0)	7 (35.0)

Adverse events were coded using MedDRA 26.0 NCI CTCAE version 5.0 or ASTCT Consensus Grading for toxicity grades

*MedDRA* Medical Dictionary for Regulatory Activities

^a^Grade 5 TEAE occurring during the treatment period; *n* (%), number and percentage of patients with at least one TEAE

### Biomarker analysis

**Fig. 2:**
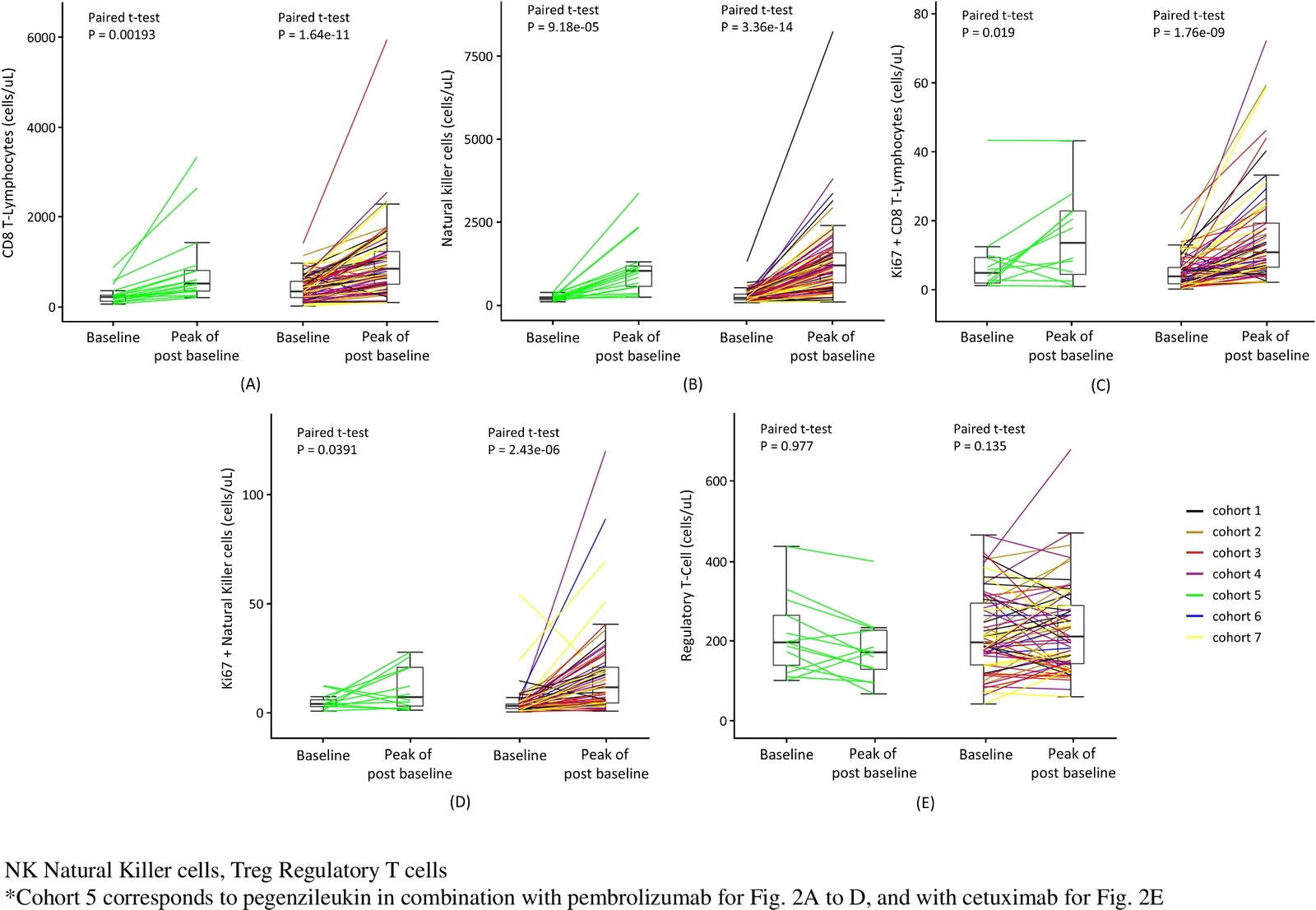
Boxplot showing the modulation of immune cells from baseline. CD8 T-lymphocytes (**A**), NK cells (**B**), Ki67^+^ CD8 T-lymphocytes (**C**), Ki67^+^ NK cells (**D**), and Treg cells (**E**)*. *NK* Natural Killer cells, *Treg* Regulatory T cells. *Cohort 5 corresponds to pegenzileukin in combination with pembrolizumab for Fig. 2A to D, and with cetuximab for Fig. 2E

Robust expansion of CD8^+^ T cells, CD8 Ki67^+^ T cells, NK cells, and NK Ki67^+^ cells was observed in patients following treatment with pegenzileukin in conjunction with pembrolizumab or cetuximab. The boxplots depicting the modulation of these cell populations show expansion at the peak value from cycle 1 to cycle 2 (Fig. 2A–D). No significant modulation of regulatory T cells was observed following treatment with pegenzileukin in combination with pembrolizumab or cetuximab (Fig. 2E).

Increased levels of cytokines (IFN-γ, IL-5, IL-6, IL-8, and TNF-α) were observed on treatment with pegenzileukin and pembrolizumab or cetuximab in patients with or without cytokine release syndrome (Supplementary Table S5). Due to the limited sample size of the tumor tissue biopsies analyzed by immunohistochemistry, it is difficult to demonstrate a statistically significant association in biomarker analysis. The immunohistochemistry analysis did not reveal a clear correlation between PD-1 or CD8 T-cell expression and reduction in tumor burden. Additionally, a very low number of NK cells were observed in the tumor microenvironment (Supplementary Fig. S2).

## Discussion

This study evaluated the efficacy and safety of pegenzileukin in combination with pembrolizumab or cetuximab across various cohorts of patients with advanced metastatic GI cancer. The preliminary assessment revealed that the observed antitumor activity at the current dose and schedule of Q3W of pegenzileukin in combination with either pembrolizumab or cetuximab was lower than anticipated. The modest efficacy observed in this study, highlights the need for further optimization of treatment strategies in these advanced GI cancer settings. Nevertheless, the safety profile remained generally manageable, with no new safety concerns identified relative to those previously reported for this compound [9, 15].

The efficacy results in terms of the ORR observed in this study are in line with those reported in the HAMMER phase 1/2 trial, where pegenzileukin demonstrated notable antitumor activity both as a monotherapy and in combination with pembrolizumab or cetuximab. In the HAMMER trial, the ORR varied across different tumor types, with notable responses in patients with melanoma and colorectal cancer [9, 15].

Recent studies involving PD-1 inhibitors, such as nivolumab and pembrolizumab, have demonstrated significant efficacy across multiple cancer types [19, 29]. Nivolumab, either alone or in combination with chemotherapy, enhanced survival rates and delayed disease progression in patients with advanced stomach and GEJ adenocarcinoma [19]. Similarly, pembrolizumab monotherapy demonstrated a clinically meaningful survival benefit and notable antitumor activity with a PD-L1 combined positive score (CPS) of ≥ 10 in patients with gastric or GEJ cancer from KEYNOTE-059, KEYNOTE-061, and KEYNOTE-062 [29]. The KEYNOTE-859 trial demonstrated significant improvements in overall survival, PFS, and overall response rate with pembrolizumab plus chemotherapy versus chemotherapy alone in patients with HER2-negative gastric or GEJ cancer, regardless of PD-L1 expression status [27]. Similarly, the KEYNOTE-811 trial showed that adding pembrolizumab to trastuzumab and chemotherapy significantly improved PFS in patients with HER2-positive gastric or GEJ cancer [14].

Pembrolizumab monotherapy has been approved for ESCC and GC/GEJ adenocarcinoma. Moreover, its benefits are predominantly observed in patients whose tumors express PD-L1, with a CPS of ≥ 1 or ≥ 10. The overall response rates for these subgroups were 16% (CPS ≥ 1) and 22% (CPS ≥ 10), respectively [11, 16]. In the phase 3 KEYNOTE-590 study, the median overall survival was 12.4 months with pembrolizumab plus chemotherapy versus 9.8 months with chemotherapy alone, and the ORR was 45.0% versus 29.3%, respectively. These benefits were particularly pronounced in patients with PD-L1 CPS ≥ 10 and in those with squamous cell histology. However, when pembrolizumab combined with pegenzileukin in our study, the ORR varied across all cohorts, with the highest (20%) in the ESCC cohort. Pembrolizumab has not demonstrated clear efficacy so far as monotherapy in MSS/MMR-proficient colorectal cancer or in HCC after progression on prior PD-1 therapy. In this study, prior treatment history varied across the cohorts.

In the GC cohort, 30.5% and 40.7% of patients had received prior immunotherapy and targeted therapy, respectively. Notably, all patients (100%) in the ESCC cohorts had previous exposure to immunotherapy; however, none had prior exposure to targeted therapy. Ninety-five percent of patients with HCC had received both immunotherapy and targeted therapy. Moreover, CRC Cohorts 4 and 5 had no prior immunotherapy, but 98.1% had received targeted therapy. The response rate observed in this study was comparatively lower, likely due to the heterogeneity in prior treatment exposures across the diverse patient population.

The safety profile of pegenzileukin combination observed in this study is consistent with the known safety profiles of pegenzileukin, pembrolizumab, and cetuximab, associated with T-cell activation. The most frequently reported TEAEs were asthenia, infusion-related reactions, and cytokine release syndrome, which were also commonly reported in the HAMMER trial [15]. The incidence of grade ≥ 3 adverse events was relatively low. In addition, no events of vascular leak syndrome were reported in this study.

The assessment of biomarkers revealed robust expansion of CD8 T cells and NK cells, with an upregulation of proliferating Ki67^+^ CD8 T cells and NK cells. Furthermore, the combination of pegenzileukin and pembrolizumab or cetuximab resulted in expansion of CD8 T cells and NK cells, without an increase in CD4 Tregs or eosinophil counts (a marker of potential vascular leak syndrome), in patients with advanced and metastatic solid tumors [21]. These findings are in line with preclinical evidence and support the mechanism of action of pegenzileukin, which selectively blocks the IL-2Rα chain while retaining engagement with IL-2Rβγ [21, 24]. Despite achieving significant peripheral CD8 + T cell expansion, limited clinical response in terms of tumor shrinkage was noted. Probably, this could be due to inadequate infiltration to tumor sites due to physical and biochemical trafficking barriers, or systemic immune activation alone might be insufficient to overcome the immunosuppressive tumor microenvironment in advanced GI cancers.

This study has a few limitations, including the limited number of patients evaluated in an open-label, non-randomized, uncontrolled trial. Additionally, early termination of the study and the relatively short treatment period further limit the interpretation of the findings. Moreover, heterogenous population (as received different prior treatment lines), lack of paired biopsies to assess intratumoral immune cell activation and lack of statistical power also restricts the interpretation of the results, making it challenging to draw definitive conclusions about the efficacy of pegenzileukin in combination with pembrolizumab or cetuximab. Further, dose optimization in future studies is warranted to evaluate if it enhances therapeutic efficacy. The lower number of responders in each cohort limits the interpretability of the secondary endpoints, such as TTR. All these limitations affect the inferences about the treatment’s effectiveness and its impact on various outcome measures.

In conclusion, pegenzileukin treatment demonstrated promising biomarker data that support pegenzileukin’s mechanism of action, particularly the upregulation of CD8 T cells and NK cells. However, despite these pharmacodynamic effects, combination of pegenzileukin with either pembrolizumab or cetuximab yielded limited antitumor activity across the GI cancer cohorts studied. We did not observe any biomarkers demonstrating significant correlation to the clinical response. Nevertheless, the safety profile was manageable. The tolerability of this approach supports its potential inclusion in future therapeutic development strategies aimed at enhancing clinical response.

## Supplementary Information

Below is the link to the electronic supplementary material.

ESM 1 Supplementary Material 1 (DOCX 691 KB)
