# Supplementary material for: Evaluating pegenzileukin (SAR444245/THOR-707) in combination with pembrolizumab or cetuximab in advanced metastatic gastrointestinal cancer: the PEGATHOR phase 2 study
Source: Invest New Drugs. 2026 Jun 22;44(5):359–70. doi: 10.1007/s10637-026-01622-2 (PMC13736307; doi:10.1007/s10637-026-01622-2)
Supplement: Supplementary file 1 [file 10637_2026_1622_MOESM1_ESM.docx]

**Article type: Research Articles**

**Evaluating Pegenzileukin (SAR444245/THOR-707) in Combination with Pembrolizumab or Cetuximab in Advanced Metastatic Gastrointestinal Cancer: The PEGATHOR Phase 2 Study**

**Supplementary Tables**

Table S1. Overview of study cohorts

| **Cohort** | **Study intervention** | **Disease** |
| --- | --- | --- |
| 1 | Pegenzileukin + pembrolizumab as 1-3L therapy | GC or Siewert Type 2 and 3 GEJ, CPS ≥1, PD-1/PD-L1 naïve |
| 2 | Pegenzileukin + pembrolizumab as 1-3L therapy | GC or Siewert Type 2 and 3 GEJ, CPS <1, PD-1/PD-L1 naïve |
| 3 | Pegenzileukin + pembrolizumab as 2-4L therapy | GC or Siewert Type 2 and 3 GEJ, post PD-1/PD-1 |
| 4 | Pegenzileukin + pembrolizumab as 3-6L therapy | CRC, any *RAS*, PD-1/PD-L1 naïve |
| 5 | Pegenzileukin + cetuximab as 3-6L therapy | CRC, *RAS* wild type |
| 6 | Pegenzileukin + pembrolizumab as 2/3L therapy | ESCC, post PD-1/PD-L1 |
| 7 | Pegenzileukin + pembrolizumab as 2/3L therapy | HCC, post PD-1/PD-L1 |

1-3L, first-line to third-line; 2-4L, second-line to fourth-line; 3-6L, third-line to sixth-line; 2/3L, Second- or third-line; CPS, combined positive score; CRC, colorectal carcinoma; ESCC, esophageal squamous cell carcinoma; GC, gastric cancer; GEJ, gastro-esophageal junction adenocarcinoma; HCC, hepatocellular carcinoma; PD-1, programmed cell death 1; PD-L1, Programmed cell death-ligand 1.

**Table S2.** Disposition of patients

| n (%) | Cohort 1 Pegenzileukin  24 µg/kg + pembrolizumab (N=22) | Cohort 2 Pegenzileukin  24 µg/kg + pembrolizumab (N=19) | Cohort 3 Pegenzileukin  24 µg/kg + pembrolizumab (N=18) | Cohort 4 Pegenzileukin  24 µg/kg + pembrolizumab (N=30) | Cohort 5 Pegenzileukin  24 µg/kg + cetuximab  (N=24) | Cohort 6 Pegenzileukin  24 µg/kg + pembrolizumab  (N=5) | Cohort 7 Pegenzileukin  24 µg/kg + pembrolizumab (N=20) | |
| --- | --- | --- | --- | --- | --- | --- | --- | --- |
| Enrolled and exposed | 22 (100) | 19 (100) | 18 (100) | 30 (100) | 24 (100) | 5 (100) | 20 (100) | |
| On intervention until data lock point | 2 (9.1) | 0 | 0 | 0 | 0 | 0 | 1 (5.0) | |
| Did not complete the study treatment period | 20 (90.9) | 19 (100) | 18 (100) | 30 (100) | 24 (100) | 5 (100) | 19 (95.0) | |
| Reason for permanent full intervention discontinuation | | | | | | | |  |
| Adverse event | 6 (27.3) | 3 (15.8) | 1 (5.6) | 2 (6.7) | 0 | 0 | 3 (15.0) | |
| Progressive disease | 13 (59.1) | 13 (68.4) | 17 (94.4) | 28 (93.3) | 24 (100) | 3 (60.0) | 16 (80.0) | |
| Poor compliance to protocol | 0 | 0 | 0 | 0 | 0 | 0 | 0 | |
| Withdrawal by subject | 0 | 2 (10.5) | 0 | 0 | 0 | 1 (20.0) | 0 | |
| Other | 1 (4.5) | 1 (5.3) | 0 | 0 | 0 | 1 (20.0) | 0 | |
| Completed the study period^a^ | 0 | 1 (5.3) | 4 (22.2) | 1 (3.3) | 5 (20.8) | 4 (80.0) | 7 (35.0) | |
| Did not complete the study period | 19 (86.4) | 18 (94.7) | 13 (72.2) | 29 (96.7) | 19 (79.2) | 1 (20.0) | 11 (55.0) | |
| Patients on pegenzileukin until data lock point | 2 (9.1) | 0 | 0 | 0 | 0 | 0 | 1 (5.0) | |
| Patients with permanent partial discontinuation of Pegenzileukin | 0 | 0 | 2 (11.1) | 0 | 1 (4.2) | 0 | 0 | |
| Patients on pembrolizumab/cetuximab until data lock point | 2 (9.1) | 0 | 0 | 0 | 0 | 0 | 1 (5.0) | |
| Patients with permanent partial discontinuation of pembrolizumab or cetuximab | 0 | 0 | 0 | 0 | 0 | 0 | 0 | |

^a^The study period included the follow up period after treatment discontinuation.

Permanent full intervention discontinuation was defined as the discontinuation of all study drugs in the combination. When all study drugs were not discontinued at the same time, the reason for permanent full discontinuation was the justification for the discontinuation of the last study drug.

**Table S3.** Most frequent TEAEs (>20%)

| n (%) | Cohort 1 Pegenzileukin  24 µg/kg + pembrolizumab (N=22) | | Cohort 2 Pegenzileukin  24 µg/kg + pembrolizumab (N=19) | | Cohort 3 Pegenzileukin  24 µg/kg + pembrolizumab (N=18) | | Cohort 4 Pegenzileukin  24 µg/kg + pembrolizumab (N=30) | | Cohort 5 Pegenzileukin  24 µg/kg + cetuximab  (N=24) | | Cohort 6 Pegenzileukin  24 µg/kg + pembrolizumab  (N=5) | | Cohort 7 Pegenzileukin  24 µg/kg + pembrolizumab (N=20) | |
| --- | --- | --- | --- | --- | --- | --- | --- | --- | --- | --- | --- | --- | --- | --- |
| Most frequent TEAE (≥20% in any arm by PT) | All grades | Grade ≥3 | All grades | Grade ≥3 | All grades | Grade ≥3 | All grades | Grade ≥3 | All grades | Grade ≥3 | All grades | Grade ≥3 | All grades | Grade ≥3 |
| COVID-19 | 5 (22.7) | 1 (4.5) | 3 (15.8) | 0 | 0 | 0 | 2 (6.7) | 0 | 2  (8.3) | 0 | 0 | 0 | 2 (10.0) | 0 |
| Cytokine release syndrome | 3 (13.6) | 0 | 2 (10.5) | 0 | 5 (27.8) | 1 (5.6) | 4 (13.3) | 0 | 3 (12.5) | 0 | 2 (40.0) | 0 | 5 (25.0) | 0 |
| Decreased appetite | 5 (22.7) | 0 | 10 (52.6) | 3 (15.8) | 7 (38.9) | 1 (5.6) | 8 (26.7) | 0 | 7 (29.2) | 1 (4.2) | 1 (20.0) | 0 | 3 (15.0) | 0 |
| Insomnia | 1 (4.5) | 0 | 5 (26.3) | 0 | 0 | 0 | 2 (6.7) | 0 | 1 (4.2) | 0 | **-** | **-** | **-** | **-** |
| Dyspnoea | 1 (4.5) | 0 | 4 (21.1) | 1 (5.3) | 3 (16.7) | 0 | 2 (6.7) | 0 | 3 (12.5) | 0 | 1 (20.0) | 0 | 1 (5.0) | 0 |
| Nausea | 4 (18.2) | 0 | 9 (47.4) | 0 | 6 (33.3) | 0 | 11 (36.7) | 0 | 7 (29.2) | 0 | 1 (20.0) | 0 | 7 (35.0) | 0 |
| Diarrhoea | 5 (22.7) | 0 | 7 (36.8) | 0 | 4 (22.2) | 0 | 6 (20.0) | 0 | 7 (29.2) | 0 | 2 (40.0) | 0 | 4 (20.0) | 0 |
| Constipation | 4 (18.2) | 0 | 5 (26.3) | 0 | 3 (16.7) | 0 | 6 (20.0) | 0 | 5 (20.8) | 0 | 0 | 0 | 1 (5.0) | 0 |
| Vomiting | 2 (9.1) | 0 | 4 (21.1) | 0 | 4 (22.2) | 1 (5.6) | 9 (30.0) | 0 | 6 (25.0) | 0 | 1 (20.0) | 0 | 2 (10.0) | 0 |
| Asthenia | 11 (50.0) | 1 (4.5) | 4 (21.1) | 1 (5.3) | 7 (38.9) | 3 (16.7) | 16 (53.3) | 1 (3.3) | 8 (33.3) | 0 | 3 (60.0) | 0 | 9 (45.0) | 2 (10.0) |
| Influenza-like illness | 4 (18.2) | 0 | 5 (26.3) | 0 | 5 (27.8) | 0 | 17 (56.7) | 1 (3.3) | 5 (20.8) | 0 | 1 (20.0) | 0 | 6 (30.0) | 0 |
| Pyrexia | 4 (18.2) | 0 | 4 (21.1) | 0 | 5 (27.8) | 0 | 5 (16.7) | 0 | 3 (12.5) | 0 | 0 | 0 | 4 (20.0) | 0 |
| Fatigue | 2 (9.1) | 0 | 6 (31.6) | 2 (10.5) | 3 (16.7) | 0 | 3 (10.0) | 0 | 5 (20.8) | 0 | 0 | 0 | 3 (15.0) | 0 |
| Disease progression | 4 (18.2) | 4 (18.2) | 4 (21.1) | 4 (21.1) | 1 (5.6) | 1 (5.6) | 1 (3.3) | 1 (3.3) | 5 (20.8) | 5 (20.8) | **-** | **-** | **-** | **-** |
| Oedema peripheral | 0 | 0 | 5 (26.3) | 0 | 1 (5.6) | 0 | 2 (6.7) | 0 | 2 (8.3) | 0 | 0 | 0 | 3 (15.0) | 0 |
| Weight decreased | 0 | 0 | 4 (21.1) | 0 | 2 (11.1) | 0 | 4 (13.3) | 0 | 0 | 0 | **-** | **-** | **-** | **-** |
| Infusion-related reaction | 5 (22.7) | 0 | 8 (42.1) | 1 (5.3) | 8 (44.4) | 2 (11.1) | 13 (43.3) | 1 (3.3) | 11 (45.8) | 0 | 2 (40.0) | 0 | 6 (30.0) | 0 |
| Folliculitis | **-** | **-** | **-** | **-** | **-** | **-** | 0 | 0 | 6 (25.0) | 0 | **-** | **-** | **-** | **-** |
| Abdominal pain | 3 (13.6) | 2 (9.1) | 1 (5.3) | 1 (5.3) | 2 (11.1) | 0 | 9 (30.0) | 2 (6.7) | 3 (12.5) | 1 (4.2) | 0 | 0 | 2 (10.0) | 0 |
| Rash | 3 (13.6) | 0 | 0 | 0 | 2 (11.1) | 0 | 2 (6.7) | 0 | 8 (33.3) | 0 | 2 (40.0) | 0 | 1 (5.0) | 0 |
| Back pain | 3 (13.6) | 1 (4.5) | 2 (10.5) | 0 | 1 (5.6) | 0 | 6 (20.0) | 0 | 3 (12.5) | 0 | 1 (20.0) | 0 | 1 (5.0) | 0 |
| Respiratory tract infection | 1 (4.5) | 1 (4.5) | 1 (5.3) | 0 | 0 | 0 | **-** | **-** | **-** | **-** | 1 (20.0) | 0 | 0 | 0 |
| Sepsis | **-** | **-** | **-** | **-** | **-** | **-** | **-** | **-** | **-** | **-** | 1 (20.0) | 1 (20.0) | 0 | 0 |
| Urinary tract infection | 0 | 0 | 1 (5.3) | 0 | 0 | 0 | 0 | 0 | 2 (8.3) | 0 | 1 (20.0) | 0 | 0 | 0 |
| Hypothyroidism | 0 | 0 | 1 (5.3) | 0 | 0 | 0 | 1 (3.3) | 0 | 0 | 0 | 1 (20.0) | 0 | 0 | 0 |
| Hyponatraemia | 0 | 0 | 0 | 0 | 1 (5.6) | 0 | **-** | **-** | **-** | **-** | 1 (20.0) | 1 (20.0) | 0 | 0 |
| Dry eye | **-** | **-** | **-** | **-** | **-** | **-** | 0 | 0 | 1 (4.2) | 0 | 1 (20.0) | 0 | 0 | 0 |
| Eye pruritus | **-** | **-** | **-** | **-** | **-** | **-** | 1 (3.3) | 0 | 0 | 0 | 1 (20.0) | 0 | 0 | 0 |
| Atrial flutter | **-** | **-** | **-** | **-** | **-** | **-** | **-** | **-** | **-** | **-** | 1 (20.0) | 0 | 0 | 0 |
| Cough | 0 | 0 | 3 (15.8) | 0 | 1 (5.6) | 0 | 5 (16.7) | 0 | 2 (8.3) | 0 | 1 (20.0) | 0 | 1 (5.0) | 0 |
| Dry mouth | 0 | 0 | 2 (10.5) | 0 | 1 (5.6) | 0 | 3 (10.0) | 0 | 0 | 0 | 2 (40.0) | 0 | 0 | 0 |
| Angular cheilitis | **-** | **-** | **-** | **-** | **-** | **-** | **-** | **-** | **-** | **-** | 1 (20.0) | 0 | 0 | 0 |
| Lip ulceration | **-** | **-** | **-** | **-** | **-** | **-** | **-** | **-** | **-** | **-** | 1 (20.0) | 0 | 0 | 0 |
| Dry skin | **-** | **-** | **-** | **-** | **-** | **-** | 1 (3.3) | 0 | 4 (16.7) | 0 | 2 (40.0) | 0 | 0 | 0 |
| Hyperhidrosis | 1 (4.5) | 0 | 0 | 0 | 0 | 0 | **-** | **-** | **-** | **-** | 1 (20.0) | 0 | 1 (5.0) | 0 |
| Rash pruritic | 0 | 0 | 0 | 0 | 1 (5.6) | 0 | 2 (6.7) | 1 (3.3) | 1 (4.2) | 0 | 1 (20.0) | 0 | 0 | 0 |

n (%)**,** number and percentage of patients with at least one TEAE.

Adverse events were coded using MedDRA 26.0 NCI CTCAE version 5.0 or ASTCT Consensus Grading for toxicity grades.

MedDRA, Medical Dictionary for Regulatory Activities; PT, preferred term.

**Table S4**. Treatment-emergent SAEs

| Treatment-emergent SAE | Cohort 1 Pegenzileukin 24 µg/kg + pembrolizumab (N=22) | | Cohort 2 Pegenzileukin 24 µg/kg + pembrolizumab (N=19) | | Cohort 3 Pegenzileukin  24 µg/kg + pembrolizumab (N=18) | | Cohort 4 Pegenzileukin  24 µg/kg + pembrolizumab (N=30) | | Cohort 5 Pegenzileukin  24 µg/kg +  cetuximab (N=24) | | Cohort 6 Pegenzileukin  24 µg/kg + pembrolizumab (N=5) | | Cohort 7 Pegenzileukin  24 µg/kg + pembrolizumab (N=20) | |
| --- | --- | --- | --- | --- | --- | --- | --- | --- | --- | --- | --- | --- | --- | --- |
| PT, n (%) | **All grades** | **Grade ≥3** | **All grades** | **Grade ≥3** | **All grades** | **Grade ≥3** | **All grades** | **Grade ≥3** | **All grades** | **Grade ≥3** | **All grades** | **Grade ≥3** | **All grades** | **Grade ≥3** |
| COVID-19 | 1 (4.5) | 1 (4.5) | 0 | 0 | 0 | 0 | 0 | 0 | 1 (4.2) | 0 | - | - | - | - |
| Respiratory tract infection | 1 (4.5) | 1 (4.5) | 0 | 0 | 0 | 0 | - | - | - | - | - | - | - | - |
| Device-related infection | - | - | - | - | - | - | 0 | 0 | 1 (4.2) | 1 (4.2) | - | - | - | - |
| Sepsis | - | - | - | - | - | - | - | - | - | - | 1 (20.0) | 1 (20.0) | 0 | 0 |
| Cellulitis | - | - | - | - | - | - | - | - | - | - | 0 | 0 | 1 (5.0) | 1 (5.0) |
| Pneumonia pneumococcal | - | - | - | - | - | - | 1 (3.3) | 1 (3.3) | 0 | 0 | - | - | - | - |
| Pneumonia | 1 (4.5) | 1 (4.5) | 0 | 0 | 0 | 0 | - | - | - | - | - | - | - | - |
| Pleural effusion | - | - | - | - | - | - | 1 (3.3) | 1 (3.3) | 0 | 0 | - | - | - | - |
| Pulmonary embolism | - | - | - | - | - | - | 1 (3.3) | 0 | 0 | 0 | - | - | - | - |
| Septic shock | 0 | 0 | 1 (5.3) | 1 (5.3) | 0 | 0 | - | - | - | - | - | - | - | - |
| Influenza-like illness | - | - | - | - | - | - | - | - | - | - | 0 | 0 | 1 (5.0) | 0 |
| Anaemia | 1 (4.5) | 1 (4.5) | 0 | 0 | 0 | 0 | - | - | - | - | - | - | - | - |
| Cytokine release syndrome | 1 (4.5) | 0 | 1 (5.3) | 0 | 2 (11.1) | 1 (5.6) | 0 | 0 | 1 (4.2) | 0 | 1 (20.0) | 0 | 4 (20.0) | 0 |
| Decreased appetite | 0 | 0 | 0 | 0 | 1 (5.6) | 1 (5.6) | - | - | - | - | - | - | - | - |
| Failure to thrive | 0 | 0 | 0 | 0 | 1 (5.6) | 1 (5.6) | - | - | - | - | - | - | - | - |
| Hypovolaemic shock | 1 (4.5) | 1 (4.5) | 0 | 0 | 0 | 0 | - | - | - | - | - | - | - | - |
| Dyspnoea | 0 | 0 | 1 (5.3) | 1 (5.3) | 1 (5.6) | 0 | - | - | - | - | - | - | - | - |
| Vomiting | 0 | 0 | 0 | 0 | 1 (5.6) | 1 (5.6) | - | - | - | - | - | - | - | - |
| Dysphagia | 1 (4.5) | 1 (4.5) | 0 | 0 | 1 (5.6) | 1 (5.6) | - | - | - | - | - | - | - | - |
| Abdominal pain | 2 (9.1) | 2 (9.1) | 0 | 0 | 0 | 0 | 2 (6.7) | 2 (6.7) | 1 (4.2) | 1 (4.2) | - | - | - | - |
| Vomiting | - | - | - | - | - | - | 0 | 0 | 1 (4.2) | 0 | - | - | - | - |
| Intestinal obstruction | 1 (4.5) | 1 (4.5) | 1 (5.3) | 1 (5.3) | 1 (5.6) | 1 (5.6) | 2 (6.7) | 2 (6.7) | 0 | 0 | - | - | - | - |
| Subileus | 1 (4.5) | 1 (4.5) | 0 | 0 | 0 | 0 | - | - | - | - | - | - | - | - |
| Gastrointestinal haemorrhage | 1 (4.5) | 1 (4.5) | 0 | 0 | 0 | 0 | 0 | 0 | 1 (4.2) | 1 (4.2) | - | - | - | - |
| Gastrointestinal obstruction | 0 | 0 | 0 | 0 | 1 (5.6) | 1 (5.6) | - | - | - | - | - | - | - | - |
| Gastrointestinal toxicity | 0 | 0 | 0 | 0 | 1 (5.6) | 0 | - | - | - | - | - | - | - | - |
| Gastric antral vascular ectasia | - | - | - | - | - | - | 0 | 0 | 1 (4.2) | 1 (4.2) | - | - | - | - |
| Intestinal pseudo-obstruction | - | - | - | - | - | - | 1 (3.3) | 1 (3.3) | 0 | 0 | - | - | - | - |
| Haematemesis | 0 | 0 | 0 | 0 | 1 (5.6) | 1 (5.6) | - | - | - | - | - | - | - | - |
| Rectal haemorrhage | - | - | - | - | - | - | 1 (3.3) | 1 (3.3) | 0 | 0 | - | - | - | - |
| Thrombocytopenia | - | - | - | - | - | - | 1 (3.3) | 1 (3.3) | 0 | 0 | - | - | - | - |
| Pancreatitis acute | 0 | 0 | 1 (5.3) | 1 (5.3) | 0 | 0 | - | - | - | - | - | - | - | - |
| Hepatomegaly | - | - | - | - | - | - | 1 (3.3) | 1 (3.3) | 0 | 0 | - | - | - | - |
| Immune-mediated hepatitis | - | - | - | - | - | - | - | - | - | - | 0 | 0 | 1 (5.0) | 0 |
| Small intestinal obstruction | 1 (4.5) | 1 (4.5) | 0 | 0 | 0 | 0 | - | - | - | - | - | - | - | - |
| Small intestinal perforation | 0 | 0 | 1 (5.3) | 1 (5.3) | 0 | 0 | - | - | - | - | - | - | - | - |
| Jaundice cholestatic | 1 (4.5) | 1 (4.5) | 0 | 0 | 0 | 0 | - | - | - | - | - | - | - | - |
| Hyperbilirubinaemia | - | - | - | - | - | - | 1 (3.3) | 1 (3.3) | 0 | 0 | - | - | - | - |
| Hypertransaminasaemia | - | - | - | - | - | - | 1 (3.3) | 1 (3.3) | 0 | 0 | - | - | - | - |
| Back pain | 1 (4.5) | 1 (4.5) | 1 (5.3) | 0 | 0 | 0 | 0 | 0 | 1 (4.2) | 0 | - | - | - | - |
| Asthenia | - | - | - | - | - | - | - | - | - | - | 0 | 0 | 1 (5.0) | 0 |
| Renal failure | 1 (4.5) | 1 (4.5) | 0 | 0 | 0 | 0 | 1 (3.3) | 0 | 0 | 0 | - | - | - | - |
| Acute kidney injury | 1 (4.5) | 1 (4.5) | 0 | 0 | 0 | 0 | - | - | - | - | - | - | - | - |
| Ureteric compression | - | - | - | - | - | - | 0 | 0 | 1 (4.2) | 0 | - | - | - | - |
| Disease progression | 4 (18.2) | 4 (18.2) | 4 (21.1) | 4 (21.1) | 1 (5.6) | 1 (5.6) | 1 (3.3) | 1 (3.3) | 5 (20.8) | 5 (20.8) | - | - | - | - |
| Chest pain | 0 | 0 | 1 (5.3) | 1 (5.3) | 0 | 0 | - | - | - | - | - | - | - | - |
| Infusion-related reaction | 1 (4.5) | 0 | 2 (10.5) | 1 (5.3) | 2 (11.1) | 1 (5.6) | 3 (10.0) | 1 (3.3) | 2 (8.3) | 0 | 1 (20.0) | 0 | 0 | 0 |
| Metastases to bone | - | - | - | - | - | - | 1 (3.3) | 0 | 0 | 0 | - | - | - | - |
| Malignant ascites | - | - | - | - | - | - | 1 (3.3) | 0 | 0 | 0 | - | - | - | - |
| Immune effector cell-associated neurotoxicity syndrome | - | - | - | - | - | - | 0 | 0 | 1 (4.2) | 1 (4.2) | - | - | - | - |
| Radiculopathy | - | - | - | - | - | - | 1 (3.3) | 1 (3.3) | 0 | 0 | - | - | - | - |
| Hip fracture | - | - | - | - | - | - | 1 (3.3) | 1 (3.3) | 0 | 0 | - | - | - | - |
| Lumbar vertebral fracture | - | - | - | - | - | - | 1 (3.3) | 1 (3.3) | 0 | 0 | - | - | - | - |

n (%), number and percentage of patients with at least one treatment-emergent SAE.

PT, preferred term.

**Table S5.** Mean cytokine levels in patients with and without CRS treated with pegenzileukin + pembrolizumab or cetuximab

|  | Cohort 1 Pegenzileukin 24 µg/kg + pembrolizumab (N=22) | | Cohort 2 Pegenzileukin 24 µg/kg + pembrolizumab (N=19) | | Cohort 3 Pegenzileukin  24 µg/kg + pembrolizumab (N=18) | | Cohort 4 Pegenzileukin  24 µg/kg + pembrolizumab (N=30) | | Cohort 5 Pegenzileukin  24 µg/kg +  cetuximab (N=24) | | Cohort 6 Pegenzileukin  24 µg/kg + pembrolizumab (N=5) | | Cohort 7 Pegenzileukin  24 µg/kg + pembrolizumab (N=20) | |
| --- | --- | --- | --- | --- | --- | --- | --- | --- | --- | --- | --- | --- | --- | --- |
|  | **No CRS**  **(N=19)** | **CRS (N=3)** | **No CRS**  **(N=17)** | **CRS**  **(N=2)** | **No CRS**  **(N=11)** | **CRS**  **(N=5)** | **No CRS**  **(N=25)** | **CRS**  **(N=4)** | **No CRS**  **(N=21)** | **CRS**  **(N=1)** | **No CRS**  **(N=15)** | **CRS**  **(N=4)** | **No CRS**  **(N=3)** | **CRS**  **(N=2)** |
| IFN-γ (pg/mL) |  |  |  |  |  |  |  |  |  |  |  |  |  |  |
| Number | 19 | 3 | 16 | 2 | 11 | 5 | 24 | 4 | 21 | 1 | 15 | 4 | 3 | 2 |
| Mean (SD) | 978.232 (707.854) | 1285.077 (872.550) | 1041.018 (878.513) | 470.571 (318.138) | 1029.485 (801.760) | 1451.498 (997.050) | 1075.197 (685.978) | 1152.395 (725.249) | 484.795 (722.565) | 2050.902 (NC) | 982.273 (730.710) | 1620.204 (1104.854) | 787.869 (11.472) | 1484.717 (542.316) |
| IL-5 (pg/mL) |  |  |  |  |  |  |  |  |  |  |  |  |  |  |
| Number | 19 | 3 | 17 | 2 | 11 | 5 | 25 | 4 | 19 | 1 | 15 | 4 | 3 | 2 |
| Mean (SD) | 3.744 (5.794) | 1.642 (1.128) | 2.898 (3.000) | 11.290 (8.246) | 8.144 (15.861) | 9.929 (12.180) | 5.944 (10.592) | 1.305 (1.776) | 1.385 (3.201) | 8.843 (NC) | 6.775 (11.121) | 3.757 (3.914) | 9.162 (1.875) | 4.540 (3.671) |
| IL-6 (pg/mL) |  |  |  |  |  |  |  |  |  |  |  |  |  |  |
| Number | 19 | 3 | 17 | 2 | 11 | 5 | 25 | 4 | 21 | 1 | 15 | 4 | 3 | 2 |
| Mean (SD) | 138.071 (216.674) | 313.544 (72.065) | 164.162 (139.811) | 437.469 (544.696) | 212.458 (147.307) | 356.102 (451.285) | 276.132 (248.593) | 221.511 (161.440) | 113.713 (189.088) | 105.303 (NC) | 187.415 (198.611) | 120.959 (46.107) | 57.654 (17.807) | 255.946 (237.364) |
| IL-8 (pg/mL) |  |  |  |  |  |  |  |  |  |  |  |  |  |  |
| Number | 18 | 3 | 17 | 1 | 11 | 5 | 25 | 4 | 21 | 1 | 15 | 4 | 3 | 2 |
| Mean (SD) | 220.076 (119.345) | 380.182 (244.059) | 389.563 (342.149) | 173.667 (NC) | 243.054 (212.478) | 190.212 (162.190) | 326.042 (273.669) | 428.693 (256.756) | 147.290 (144.245) | 136.015 (NC) | 401.534 (229.385) | 188.628 (204.534) | 108.106 (0.880) | 475.231 (79.279) |
| TNF-a (pg/mL) |  |  |  |  |  |  |  |  |  |  |  |  |  |  |
| Number | 19 | 3 | 17 | 2 | 11 | 4 | 25 | 4 | 21 | 1 | 15 | 4 | 3 | 2 |
| Mean (SD) | 12.067 (5.855) | 14.446 (4.557) | 16.808 (10.986) | 19.069 (4.353) | 14.950 (10.226) | 14.519 (6.642) | 17.046 (17.440) | 16.550 (3.490) | 8.814 (6.969) | 17.398 (NC) | 13.427 (6.517) | 19.073 (7.484) | 16.617 (6.635) | 21.150 (11.792) |

CRS, cytokine release syndrome; SD, standard deviation

**Supplementary Figures**

**Figure S1.** Study design

AE, adverse event; C, study cycle; D, study day; Q9W = every 9 weeks; Q12W = every 12 weeks; EOT, end of treatment; ORR, objective response rate; RECIST, Response Evaluation Criteria Solid Tumors.

**Figure S2.** Biomarker analysis in the tumor microenvironment and its association with clinical response

BOR, best overall response; NE, non-evaluable; PD, progressive disease; PD1, programmed cell death 1; PR, partial response; SD, stable disease.
